# SOBER-MCS: Sociability-Oriented and Battery Efficient Recruitment for Mobile Crowd-Sensing

**DOI:** 10.3390/s18051593

**Published:** 2018-05-17

**Authors:** Fazel Anjomshoa, Burak Kantarci

**Affiliations:** 1 Department of Electrical and Computer Engineering, Clarkson University, Potsdam, NY 13699, USA; anjomsm@clarkson.edu; 2 School of Electrical Engineering and Computer Science, University of Ottawa, Ottawa, ON K1N 6N5, Canada

**Keywords:** mobile crowd-sensing, behaviometrics, energy efficiency, user profiling, smart cities

## Abstract

The Internet of Things (IoT) concept is aiming at being an integral part of the next generation networking services by introducing pervasiveness and ubiquitous interconnectivity of uniquely-identifiable objects. The massive availability of personalized smart devices such as smartphones and wearables enable their penetration into the IoT ecosystem with their built-in sensors, particularly in Mobile Crowd-Sensing (MCS) campaigns. The MCS systems achieve the objectives of the large-scale non-dedicated sensing concept in the IoT if a sufficient number of participants are engaged to the collaborative data acquisition process. Therefore, user recruitment is a key challenge in MCS, which requires effective incentivization of cooperative, truthful and trustworthy users. A grand concern for the participants is the battery drain on the mobile devices. It is a known fact that battery drain in a smartphone is a function of the user activity, which can be modeled under various contexts. With this in mind, we propose a new social activity-aware recruitment policy, namely Sociability-Oriented and Battery-Efficient Recruitment for Mobile Crowd-Sensing (SOBER-MCS). SOBER-MCS uses sociability and the residual power of the participant smartphones as two primary criteria in the selection of participating devices. The former is an indicator of the participant willingness toward sensing campaigns, whereas the latter is used to prioritize personal use over crowd-sensing under critical battery levels. We use sociability profiles that were obtained in our previous work and use those values to simulate the sociability behavior of a large pool of participants in an MCS environment. Through simulations, we show that SOBER-MCS is able to introduce battery savings up to 18.5% while improving user and platform utilities by 12% and 20%, respectively.

## 1. Introduction

Smart mobile devices have become an inseparable part of everyday activities including e-business, healthcare, social interactions and entertainment [1]. Gartner’s report for 2018 forecasts smartphone sales to exceed 2.35 B by the end of the year, which is the highest growth rate in the last three years [2]. With their built-in sensing and storage capability, along with improved computing and communication capabilities, smartphones and wearables appear to be the best candidates for non-dedicated sensing campaigns, particularly the Mobile Crowd-Sensing (MCS) concept. As a reasonably new non-dedicated sensing concept, MCS empowers the utilization of built-in sensors (e.g., GPS, gyroscope, microphone, accelerometer, light and camera) on the smart mobile devices in an ad hoc, but pre-enrolled participant pool [3] to acquire and analyze data to be used in various applications such as remote healthcare [4,5,6] and environmental and traffic monitoring and management [7,8,9,10].

Participants of an MCS campaign offer multi-sensory data acquired from personalized smart devices, which normally include GPS, gyroscope, microphone, light, camera, accelerometer and wireless communication interfaces [11,12]. Data acquired from multiple participants are aggregated and further analyzed at a central platform. Data acquisition in MCS can be performed in either of two forms: participatory or opportunistic [7]. The former seeks the active involvement of participants in the sensing process, whereas the latter minimizes participant involvement since applications or devices are the decisive drivers for sensing campaigns. In both opportunistic and participatory sensing, user recruitment requires the involvement of a centralized platform that accounts for various criteria in the selection of users, as well as participant-task matching [13].

Since no upfront infrastructural investment is required, MCS can achieve effective acquisition of sensory data and leads to accurate and precise analysis if user participation is elevated. Therefore, user recruitment is a grand challenge in participatory MCS systems. Designing suitable algorithms to address the utility trade-off problem between the participants and the MCS platform has been an open issue in the literature since the MCS concept was initially coined [14]. At both the platform and user ends, performance evaluation involves optimization of incomes and costs. The income of the platform is related to the value of the sensed data, and it is defined by the Sensing as a Service (S${}^{2}$aaS) business models [15], whereas the compensation of the users for their contributions translates into the platform cost. User costs in the participation of data aggregation are basically the power drained from the batteries for sensing the tasks and cellular data usage/charges in case sensor readings are reported via a cellular network interface.

Energy consumption or battery drain, along with other concerns, is one of the main parameters that contributes to the user incentives due to the power-hungry nature of the mobile devices in the MCS environment [16]. Hence, effective incentives in the acquisition of crowd-sensed data should aim at maximizing the benefits (i.e., utility) at both the user and platform ends and minimizing energy costs at the user end [17].

The advances in data mining and machine learning enable the development of predictive models for the discovery of events, communities and knowledge [18,19]. Thus, introducing such intelligence to the MCS system could reveal behavioral patterns of users and can consequently help to ameliorate platform costs. In [20], a mobile behaviometric framework is proposed to assess users’ activity on social networking platforms and introduce sociability metrics to generate fingerprints of the online social behavior of users. Through various machine learning algorithms, various profiles and usage patterns are discovered. The discovered information in that study is invaluable for an MCS study since it can be used in user recruitment to meet the two objectives that were mentioned above.

In this article, we aim to cope with the battery drains of the participants in an MCS system. To this end, we propose a novel methodology for participant recruitment for participatory data acquisition in MCS systems by introducing a multi-context user recruitment policy into user incentives, which is based on two indicators: (i) user sociability, which denotes an indication of willingness for participation in crowd-sensing campaigns, and (ii) the energy, which denotes the residual battery level of mobile devices. The proposed scheme is called Sociability-Oriented and Battery-Efficient Recruitment for Mobile Crowd-Sensing (SOBER-MCS). The sociability-awareness in participant recruitment enforces the recruitment of active users, whereas energy-awareness enables prioritizing personal use of the participant devices in lieu of sharing built-in sensors when the residual battery level drops below a certain threshold. We run extensive simulations for the performance evaluation of SOBER-MCS and show that the multi-context user recruitment can improve battery consumption up to 18.5% while improving the user and platform utilities up to 12% and 20%, respectively.

The article is organized as follows. In Section 2, we present the related work and background on the subject. Section 3 presents the proposed recruitment model in detail. The performance evaluation of the proposed solution is presented in Section 4. Finally, Section 5 concludes the article and provides future directions.

## 2. Related Work

**Figure 1:**
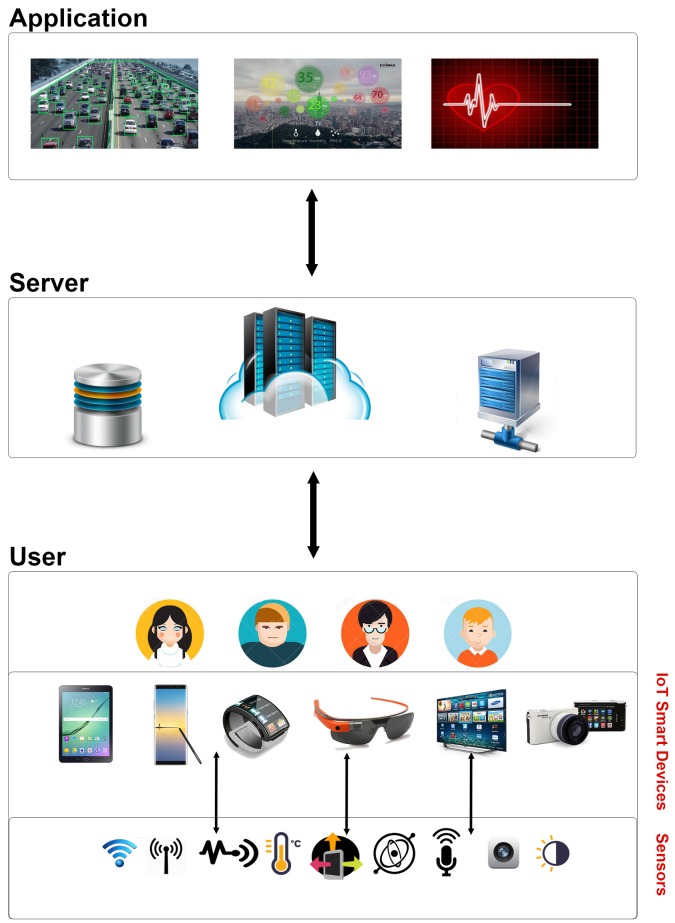
Building blocks of a typical Mobile Crowd-Sensing (MCS) system.

MCS systems are envisioned to be a part of an Internet of Things (IoT) ecosystem [21] where sensed data are acquired from mobile smart devices in a participatory or opportunistic manner. Collected data are reported to the MCS platform, which is, in fact, a sensing server located in a cloud platform, and undertakes data analytics and visualization tasks on the crowd-sensed data. Figure 1 illustrates the building blocks of a conventional MCS system.

The end user of an MCS campaign reserves a limited budget to recruit and reward participants for their contribution. Therefore, it is vital to have a solid recruitment policy in order to minimize the costs and ensure that the participants who provide useful data will be recruited for the highest possible quality of the analytics result. In particular for mission-critical applications, selecting cooperative participants that are of utmost trustworthiness is of paramount importance [22,23]. Designing the optimal solution for recruiting users and assigning tasks among selected users is a grand challenge in MCS systems. The objective of such a solution is to make a compromise between maximum user and platform utilities. Furthermore, in the presence of malicious or unreliable participants, the trustworthiness of crowd-sensed data is also critical, as it directly impacts the platform utility [24].

As battery limitation of smart mobile devices is a key concern for participants, data acquisition and user recruitment models that account for energy efficiency and energy modeling are emergent. The authors in [25] model the power consumption of a participating mobile device in an MCS application under three categories: power consumption in data transfer [19,26], power consumption in sensing [27,28] and power consumption in computation [29,30].

The study in [27] proposes an effective energy-saving scheme in the recognition of human physical activities through machine learning algorithms such as the Hidden Markov Model (HMM) and k-Nearest-Neighbors (kNN). A similar study has been carried out by the authors in [28] on wearable sensors, particularly accelerometers and gyroscopes that provide raw sensor data to Convolutional Neural Networks (DCNN). Karaliopoulos et al. [31] consider the context of opportunistic sensing systems and propose a solution to minimize the cost and improve the accuracy of sensed data by predicting user location via deterministic and stochastic mobility models [31]. Piggyback crowd-sensing is another well-known technique that can be utilized in MCS systems to ensure power efficiency [19,32]. Piggyback crowd-sensing aims at predicting the active usage/interaction patterns of the users with their mobile devices in order to upload collected data to the central platform [17]. In [32], three greedy algorithms are proposed to find the optimal number of participants who are capable of fulfilling the assigned task, whereas historical records are utilized by the research in [19] to predict future user calls and find the area of sensing coverage. He et al. [33] present a pricing scheme that is built upon a bargaining theory-based technique for assigning tasks in a time-dependent manner among the participants. In [25], an energy-efficient MCS strategy, namely the two-call-based approach, is proposed to exploit phone calls instead of mobile data plans for anonymous transmission of tasks. The first phase of two-call-based approach predicts whether a user is a potential participant for the upcoming campaigns. The second step simply leads the users with no tasks to a two-step decision making process that involves determining whether or not the system needs more sensing tasks in the first step, and in the second step, the system determines whether or not to select the corresponding user.

In [34], the authors address the problem of energy-efficient selection of MCS participants based on the maximum residual battery. The scheme ensures the quality and accuracy of sensed data based on the amount of data sensed during each task. Delay-tolerant sensing networks are other possible types of MCS systems where the sensed data are beneficial even after some delay. The authors in [35] propose a comprehensive framework that uses delay-tolerant routing protocols to report data acquisition. Security and privacy are the biggest concerns for the users; hence, the location awareness feature of mobile phones can be disabled at the time of selection. An alternate solution that effectively performs while addressing these concerns is spatial cloaking [36].

In [37], the authors propose to allocate the opportunistic MCS tasks in a context-aware manner with the ultimate goal of recruiting the most favorable users. To this end, a set of indicators keeps track of the participant-task matching similarity. Moreover, energy consumption is also considered by the same study in conjunction with user privacy, e.g., users are not enforced by the platform to grant access to their location.

J. An et al. [38] use social relations to find out the most efficient and trustworthy route from the requester of the service to the provider and vice versa. Normally, a requesting entity aims to obtain information through acquired data about a certain phenomenon, which is preferably provided by the service provider who co-exists with the requester in the same community. If a provider in the same community cannot offer the service, the task is extended to users of other overlapping communities. Collaboration among community members ensures successful service provisioning by exploiting the social ties between the users.

In [39], the authors propose a reverse auction procedure, namely MSensing, that intends to maximize both platform and user utility. MSensing was proposed as an improvement to the Local Search-Based (LSB) auction that recruits users with higher marginal contribution as the winners to maximize the platform utility. As an extension, in [40], the authors proposed a new auction-based mechanism called the IMCUauction, which follows the same rules and steps deployed in MSensing, but in the new research, two different sets of data are used for evaluating the performance of user-centric MCS platforms. While the proposed research proved is pretty comprehensive and competent in terms of computational efficiency, truthfulness, profitability and individual rationality, we believe there is still room for research that considers the energy efficiency of incentive methods, as well as the characteristics of each participant such as social links and user reputation, for those who contribute to the sensing of the surrounding phenomena.

## 3. Background

SOBER-MCS adopts MSensing auction-based user-centric incentives [39] for mobile crowd-sensing and introduces an energy-aware incentive mechanism that uses sociability-based user profiling.

In [20], we studied the problem of identifying behavioral patterns of smartphone/tablet users. In that study, social networking applications were the target platforms to mine behavioral patterns. The investigation aimed at identifying profiles, ideally, in a non-intrusive manner and verifying the smartphone users by their behavioral data. Prior to the behavioral identification, the study in [41] presented a mobile behaviometric framework to assess users’ social activities in terms of sociability metrics so as to generate behavioral signatures of the users. In the same study, the following two metrics were defined: social activity rate, which stands for the relative value of data generation originated by a user at the time of using social networking applications, and sociability factor, which is a function of the time that is spent by a user on a set of mobile social network applications. These two metrics are formulated as seen in Equations (1) and (2), which also exist in [41].

(1) $$A_{overall}^{U_{{app}_{x}}^{u}}\left(T_{k}\right)=\alpha *A_{sh}^{U_{{app}_{x}}^{u}}\left(T_{k}\right)+\left(1-\alpha \right)*A_{sh}^{U_{{app}_{x}}^{u}}\left(T_{k-1}\right)$$

(2) $${\mathrm{SF}}_{overall}^{U_{{app}_{x}}^{u}}\left(T_{k}\right)=\beta *{\mathrm{SF}}_{sh}^{U_{{app}_{x}}^{u}}\left(T_{k}\right)+\left(1-\beta \right)*{\mathrm{SF}}_{sh}^{U_{{app}_{x}}^{u}}\left(T_{k-1}\right)$$

These metrics are calculated based on the weighted sum of current and past values; therefore, the overall social activity rate ($A_{overall}^{U_{{app}_{x}}^{u}}\left(T_{k}\right)$) and overall sociability factor (${\mathrm{SF}}_{overall_{i}}^{U_{{app}_{x}}^{u}}\left(T_{k}\right)$) are used as the indicators of willingness in participation. In the equations, $T_{k}$ denotes the *k*-th short-term sociability factor (or social activity rate), and β stands for the weight factor of a corresponding social network application as proposed in [41].

In this article, we propose a novel methodology that assesses and exploits sociability metrics in the recruitment of MCS participants. The sociability metrics in MCS translate to the participant willingness of contributing to the sensing of tasks over different time windows. Therefore, the willingness and eligibility of different users for a given set of sensing tasks enable one to build cost-effective solutions for an MCS system.

## 4. Sociability-Oriented and Battery-Efficient Recruitment for Mobile Crowd-Sensing

In this section, we present our energy-efficient user recruitment scheme by using data-driven sociability metrics to incentivize users to participate in MCS by reducing their energy cost. Thus, the rest of this section presents the framework and the integration of the sociability metric with effective user recruitment.

**Figure 2:**
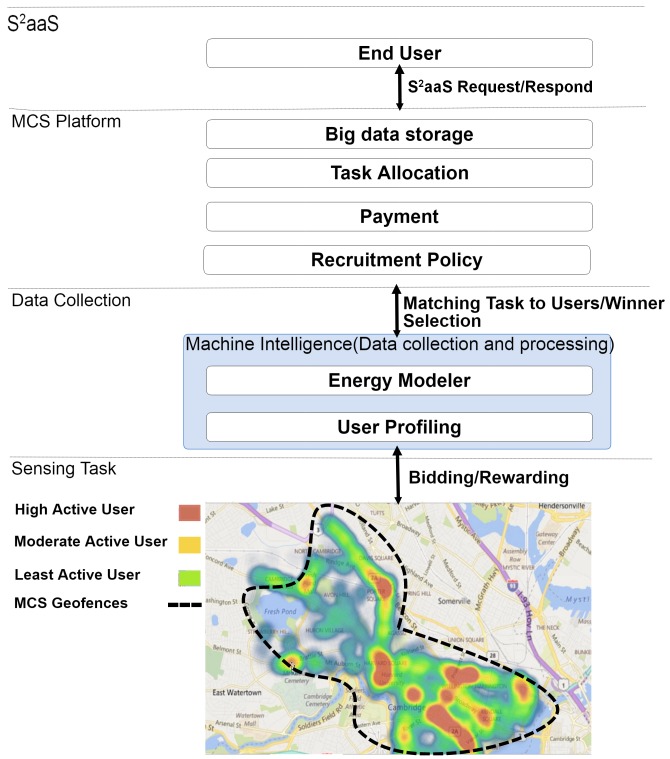
System design.

Figure 2 illustrates the proposed system in a minimalist manner. According to our proposed energy-aware incentive mechanism, participants are categorized based on their activity profiles. When a new user signs up to participate in sensing, the user’s activity rate is sent to the machine intelligence component for the classification procedure. The machine intelligence component is equipped with different machine learning models to provide high accuracy predictions regarding the user’s profile. Through different machine learning algorithms, SVM (Support Vector machine) and DBSCAN (Density-Based Spatial Clustering of Applications with Noise) and analysis of the raw data acquired from real users, three representative profiles were identified as follows: (i) highly-active users; (ii) moderately-active users; (iii) least active users. The results from the study revealed that most active users were interacting with the smartphone 70% of the time during a particular time window. The same activity rate for moderately- and least active users was found to be 50% and 15%, respectively. In accordance with their activity profile, each user has a different energy model, as he/she has different energy overhead to start sensing the task.

The MCS platform receives the task from the end user and estimates the cost of the task in terms of energy consumption based on the number of sensors that are needed to sense the task and also the duration of sensing. In this paper, five different sensors including GPS, accelerometer, microphone, light and video camera are taken into consideration. Each task involves a random combination of sensors and is also assigned a random task duration. The task and user settings are then sent to the recruitment policy component for user-task matching. The recruitment policy takes users’ profiles, locations and the residual energies of their devices into consideration and applies the reverse auction procedure to find the winners (i.e., selected participants). To ensure energy efficiency, the recruitment module introduces energy costs into the user bids, as well as the residual battery power of the participants in the selection.

### 4.1. System Architecture

**Figure 3:**
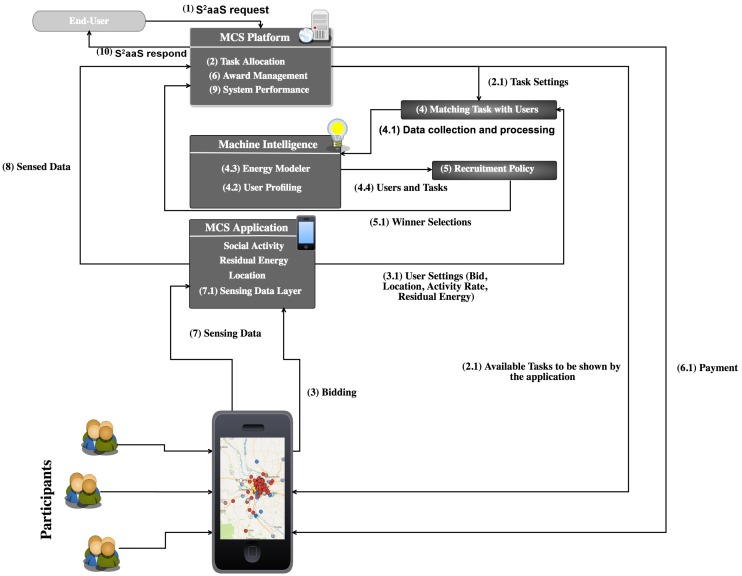
System architecture.

The system architecture of SOBER-MCS is illustrated in Figure 3. The crowd-sensing event starts with the Sensing as a Service (S${}^{2}$aaS) inquiry from the end user to the MCS platform as shown by Step (1). The platform initiates the task allocation component (2), which is responsible for assessing the task cost. Task cost is the energy consumption that is needed for sensing a task. This step involves determining the number of sensors, sensing time and the location of the requested task, as well as the estimation of sensing cost in terms of energy consumption for each task, which denotes task settings (2.1). After the evaluation of the task settings, the platform communicates with the users’ mobile application to share the task settings. Upon receiving the task settings, users start bidding on the available tasks (3). As the MCS platform builds its recruitment strategy, it collects the bids from the users as users’ bids considering the energy consumption required for sensing a task. Following this step, the bidding announcement along with the user’s profile, residual battery level of the device and the location of the user are shared with the MCS platform (3.1). The platform sends collected information from all available users and initiates the matching task with the users’ component (4). In this step, the data collected from users and information about the tasks are sent to the machine intelligence component to select the winners (4.1). Machine intelligence has two main components: user profiling (4.2) and energy modeler (4.3). The former predicts user activity, and according to the activity, the user is assigned to one of the three user profiles: (i) highly-active; (ii) moderately-active; (iii) least active. The latter is responsible for building the energy efficiency-based recruitment model based on the information from the user profiling component and task setting.

The energy modeler takes the bids for each task into consideration and determines the value of the task, the distance of users to the task, the residual energy of selected users’ devices and the energy overhead of the users based on their profiles. This information is then sent to the recruitment policy for the final step, which is the appointment of the winners of the reverse auction procedure (fifth step in Figure 3). The proposed recruitment policy estimates the energy cost of sensing tasks along with the residual energy of user devices. Furthermore, the recruitment policy also aims to balance the total energy consumption by discarding users with the remaining battery level below a threshold, i.e., minimum allowed residual energy. The threshold is motivated by excluding high risk users that are unlikely to accomplish the allocated task due to low battery level. The motivation behind introducing the energy threshold is to prevent complete battery drain in the devices of high risk users due to the assignment of a new sensing task. Besides leading to the battery drains and their consequences, the MCS platform is likely to end up experiencing degraded utility for recruiting soon to be off crowd-sensors instead of more appropriate candidates.

The list of winners are then sent to the MCS platform. The award management component of the platform handles the payment procedure (6.1). It is worth noting that this step denotes “budgeting of the payment”, not necessarily the actual transaction. The transaction can happen anytime after the sensed data have been received. The winners send the sensing data through the MCS application (7), which delivers the sensed data to the server. Upon receiving the results, the MCS platform calls system performance (9) to evaluate the results and analyze the system performance metrics.

### 4.2. Recruitment Algorithm

This paper proposes a new participant recruitment approach, SOBER-MCS, that exploits sociability metrics of users to recruit them in a cost-effective manner and introducing energy-awareness into the sociability-awareness to ensure energy efficiency for the participant smartphones. At the core of SOBER-MCS lies the recruitment algorithm. Before proceeding with the details of the algorithm, Table 1 presents the notation used in this article to define the theoretical foundations of the proposal.

As illustrated in Figure 3, upon the arrival of a sensing task, the MCS framework estimates the cost of the task in terms of potential energy consumption and shares this value with the users through the task package. Bidding starts upon receiving the task packages, and user bids are passed to the recruitment policy. The aim of the recruitment policy is to select the winners for the available task by aiming at minimum average battery drain for all participants due to participation in the MCS campaign.

At the beginning, the system evaluates users’ sociability using TrackMaison, which denotes a metric to estimate the participants’ willingness to participate in sensing tasks. Sociability ranges from 0%–100% percent, and a higher percentage denotes a higher willingness for participation. TrackMaison acquires built-in sensors’ data and statistical data regarding interaction patterns with multiple social network applications on smart mobile devices. TrackMaison then analyses the raw data and builds user profiles based on users’ social activities using different machine learning algorithms.

In the proposed sociability-aware recruitment policy, the MCS platform can predict the availability/willingness of participants by learning their historical data and estimates the energy requirement for the sensing task based on the task settings. Thus, the MCS platform can reduce the energy consumption due to participation by assigning the tasks to users that minimize the cost of participating in the MCS campaign.

### 4.3. User Profiling

**Figure 4:**
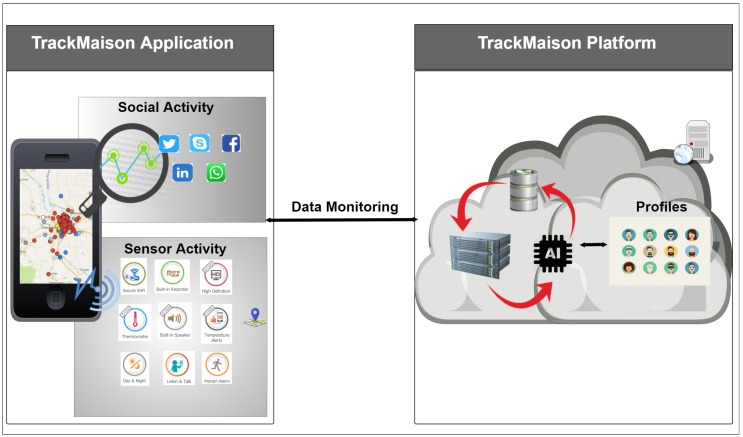
Machine intelligence module that uses the TrackMaison framework in [20].

User profiling involves the assessment of a user’s interest domain through continuous monitoring and analytics over feature sets extracted from the acquired data [42]. User profiling has a crucial role in the proposed system architecture. For this study, we use the TrackMaison framework, which is presented in [41] to model user social activity in the MCS environment. As presented in Figure 4, it consists of two core components: the back-end cloud server undertakes the storage facility for raw data, analysis functionality for the aggregated data and machine learning models for user profiling; the front-end Java application is in charge of monitoring the interaction patterns between the user and the smartphone, as well as the transmission of session data to the cloud server for analysis.

**Figure 5:**
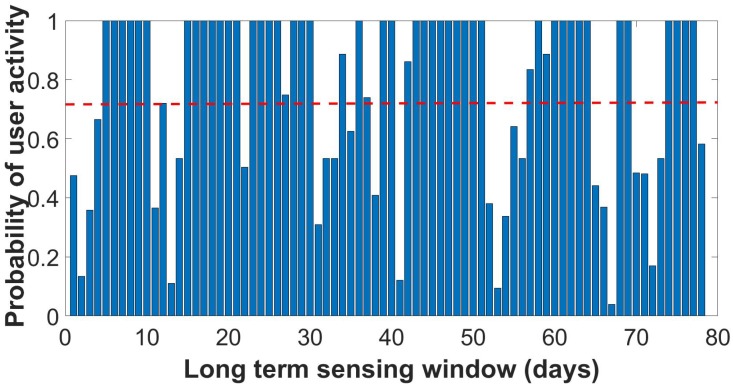
Probability of the selected high active user during sensing time window.

**Figure 6:**
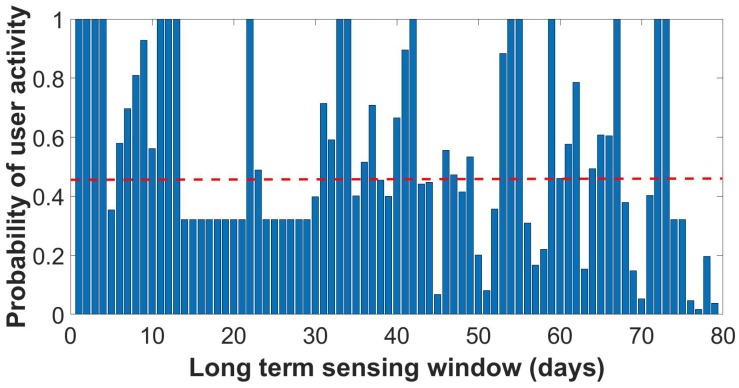
Probability of the selected moderate active user during sensing time window.

**Figure 7:**
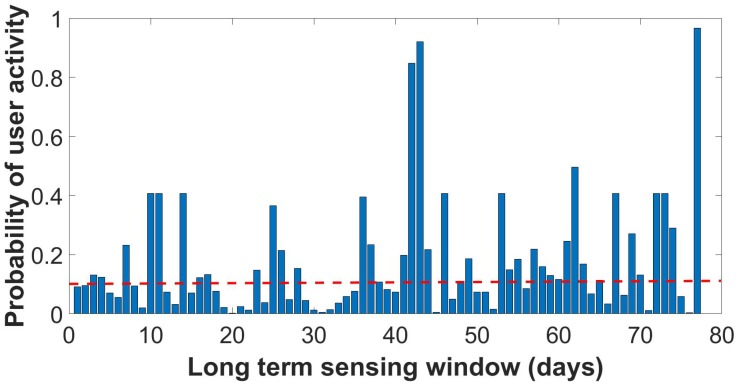
Probability of the selected least active user during sensing time window.

In the TrackMaison framework, the frond-end Java application continuously monitors user activity in five popular social network applications (i.e., Facebook, Twitter, LinkedIn, WhatsApp and Skype) along with built-in mobile sensors such as GPS. It is worth noting that the list of social network applications can be extended and/or modified; however, investigating the impact of different applications on user profiling is out of scope of this article. TrackMaison defines the following social behavior metrics: the social activity rate, which is an indicator of the data usage of the smartphone on a particular application, and the sociability factor, which indicates the time spent by a user on a particular application. Figure 5, Figure 6 and Figure 7 depict the probability distribution of the activeness of selected users over the sensing time window for the three types of users. These figures were not presented in previous studies.

By using different machine learning algorithm, SVM and DBSCAN in this study, three representative user profiles were identified from the real data traces. The highly-active user actively interacts with the device ≈70% of the monitored time window. In other words, when a highly-active user is assigned a sensing task, only 30% of the time does he/she need to prepare his/her smartphones to start sensing the task, which translates into energy overhead. The second type of user profile is the moderately-active user who actively interacts with the device ≈50% of the monitored time window. The last type of sociability profile is the least active user type who actively interacts with the device ≈15% of the monitored time window, which translates into 85% energy overhead for the user to start sensing the tasks.

### 4.4. Task Allocation

The task allocation is illustrated in Figure 3. Upon the description of a sensing task, the MCS platform estimates the energy consumption of the task by considering the number of sensors that are needed to sense the task and the sensing time. This information along with the location of the task is sent to the participants’ smart devices.

The energy consumption of task *j* ($E_{t_{j}}$) is a function of the power consumed by the task *j* ($P_{t_{j}}$) during the sensing time of the task *j* ($T_{j}$) as formulated in Equation (3). Each task utilizes a random combination of up to Φ different sensors. The power consumption for task *j* using different sensors is calculated as the summation of each sensor power that is needed for task *j* as shown in Equation (4) where $\Phi_{j}$ denotes the number of utilized sensors by task *j* and can be defined as $1\leq \Phi_{j}\leq \Phi$.

(3) $$E_{t_{j}}=\left(P_{t_{j}}\right)*T_{j}$$

(4) $$P_{t_{j}}=\sum_{s=1}^{\Phi_{j}}P_{t_{j}}^{SS_{s}}$$

Upon the estimation of the task cost via task settings (i.e., sensors’ energy consumption and task duration), the participants are notified of the task description via the MCS application. Receipt of the task description at a user’s mobile device triggers the user’s bidding for the tasks. The MCS app sends the bid of the user, which consists of the following fields: bid (i.e., sensing cost), the location of the user and the sociability rate in order for the MCS platform to assess the user’s willingness prior to user-task matching. The energy modeler is the first component called by the MCS platform immediately after the receipt of user bids. The energy modeler component is explained in the next subsection.

#### 4.4.1. Energy Modeler

The energy modeler is in charge of calculating the sensing cost based on the user’s profile and bid. Once the social activity rate and sociability factor of a user are received from the user profiling module, Equation (5) is used by the energy module to assess the energy cost of task *j* by using Equation (5):

(5) $$E_{w_{j}}^{u_{i}}=P_{t_{j}}^{u_{i}}*T_{j}+P_{O_{j}}^{u_{i}}*T_{prep}^{j}$$

As noted in Section 4.2, $P_{O_{j}}^{u_{i}}$ stands for the overhead for user *i*, which is simply the power cost for user *i* to prepare his/her device to start sensing the task *j*. This includes unlocking the phone, which involves the screen backlight, and turning on the sensor(s). The overhead contributed to the energy consumption of user *i* to sense the task *j* itself ($P_{t_{j}}^{u_{i}}*T_{j}$). In the equation, $T_{prep}^{j}$ is the time needed to prepare the smartphone to sense task *j*. Relying on the fact that users with a higher sociability factor are expected to be more active during the task assignment, the built-in sensors in their smartphones are likely to be already in use. Thus, the higher the sociability factor, the lesser the energy overhead for sensing.

#### 4.4.2. Winner Selection

**Figure 8:**
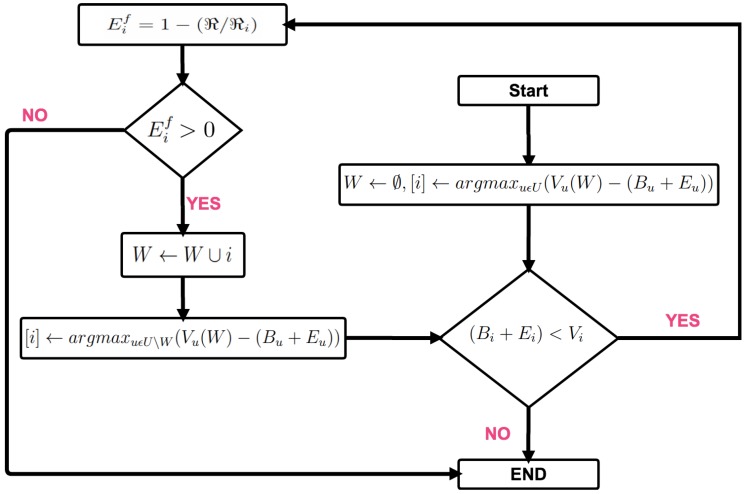
Winner selection in user recruitment.

As illustrated in Figure 3, the recruitment policy consists of two consecutive steps: (i) winner selection (i.e., Step 5 in the figure); (ii) payment determination (i.e., Step 6 in the figure). Figure 8 presents the winner selection procedure. Briefly, the winner selection relies on selecting the users whose marginal contribution are higher than their bids. To this end, in the assessment of the marginal contribution of a user, the sensing cost of users is estimated by two separate metrics: ordinary cost ($B_{u}$) and energy cost ($E_{u}$). Ordinary cost ($B_{u}$) is an indicator of user discomfort such as security, energy overheads and WiFi/mobile data plan. On the other hand, energy cost ($E_{u}$) represents the energy consumed by the mobile device to participate in an upcoming sensing campaign. The marginal value of user *i* is formulated as the difference between the value of the recruited population before and after recruiting that user as formulated in Equation (6). As shown in Figure 8, in every iteration, the platform aims at finding a user that maximizes the difference between their sensing cost (i.e., bid) and marginal value (formulated as $V_{i}-\left(B_{i}+E_{i}\right)$). This difference denotes the marginal contribution from user *i* to the recruited crowd population. If the marginal contribution of user *i* is positive ($\left(B_{i}+E_{i}\right)>V_{i}$), the MCS platform calculates the energy factor ($E_{i}^{f}$) for the user before proceeding with appointing the user as a winner. $E_{i}^{f}$ is used to balance the overall energy consumption between users with respect to the minimum residual energy, *ℜ*, that is set by the platform. Mobile devices with residual battery level (${ℜ}_{i}$) below *ℜ* are discarded by the platform, whereas the others are added to the winners’ set. Thus, the MCS platform continues to add smart devices until their marginal contribution and energy factor turn non-positive.

(6) $$V_{u}\left(W\right)=V\left(W\cup \left\{u\right\}\right)-V\left(W\right)|u\;\epsilon \;U$$

As seen in the figure, winner selection is followed by payment determination. Our payment determination procedure adopts the payment step in [39]. Thus, each winner *i* is temporarily removed from the participant list ($U^{\prime }⇐U\backslash \left\{i\right\}$), and over the rest of the participant list ($U^{\prime }$), the maximum hypothetical sensing value of another user ($j\in U^{\prime },j\neq i$) is sought, which would still result in user *i* if the user were a member of $U^{\prime }$.

## 5. Performance Evaluation

In this section, we evaluate the performance of SOBER-MCS by using the sociability profiles. We adopt the MCS simulation environment in [43] and use MSensing as our benchmark.

### 5.1. Simulation Setup

We consider the availability of five built-in sensors in smartphones: GPS, accelerometer, sound level, light sensor and camera. The power consumption of each sensor is presented in Table 2 based on the values in the literature [44,45]. It is assumed that the sensing task time is following a Gaussian normal distribution between 1 and 5 min with a mean of 3 min. The time needed to prepare a smartphone is generated randomly between 1 and 5 s.

According to [46], the power consumption of the preparation of the phone would be approximately 80 mW. With this in mind and by considering user profiles, we define the power overhead as described in Table 3 for each user profile. In more detail, as a highly-active user is active during 70% of the sensing task time, their smartphone needs 30% of the task time window to prepare for sensing, which translates to power overhead. Thus, for highly-active users, the power overhead consumption would be 0.30 × 80 mW = 24 mW. The activity rates for the moderately- and least active users are 50% and 15%, respectively. Similarly, their power overhead would be calculated as 0.5 × 80 mW = 40 mW for moderately-active users and 0.85 × 80 mW = 68 mW for the least active ones. The power overhead of user *i* for task *j* contributes to the task energy consumption formulation in Equation (5) as $P_{O_{j}}^{u_{i}}$.

Sensing tasks are uniformly distributed over a 1000 m × 1000 m terrain, and the users participate in sensing the tasks within a 30-unit range of their location, which is acquired by the GPS sensor in their smartphones. One thousand users are randomly labeled as highly-active participants, moderately-active participants and least active participants. The mobile devices have an initial residual energy that is calculated using historical data from real data gathered from TrackMaison. Highly-active users have 54% initial battery level, while this value for the moderate and least active users is 58% and 63%, respectively. The initial battery levels in this study are calculated by obtaining the average from the real traces collected by TrackMaison [41]. The arrival process of sensing tasks to the MCS platform is generated so as to follow a Poisson distribution with arrival rates varied between 20 tasks/min and 100 tasks/min. The duration of a sensing campaign is set to 30 min, while task durations are uniformly distributed between 1 min and 5 min. Task values are also uniformly distributed between one and seven. The results are averaged by running each simulation five times per each task arrival rate using five different seeds. The simulation settings are also summarized in Table 4.

For simulating a user-centric mobile crowd-sensing model, we use a discrete-event simulator built in Java and run extensive simulations. In addition, we assume that participants do not move and leave the incorporation of mobility models into this study to future work. It is worth noting that using either model, as simple as a random waypoint mobility [47] or social mobility model, is possible [48] in the performance study of MCS systems. The users are uniformly distributed over the 1000 m × 1000 m terrain. The payment method in [39] is adopted in this study. Thus, each winner in the winner selection step is temporarily eliminated from the set of winners, and the winners’ set is re-built. During every new build procedure, the maximum hypothetical value that would still cause the user to be removed is found, and a worthy candidate is calculated. Furthermore, we ensure that every participant receives a minimum payment equal to his/her announced bid if he/she is marked as a winner in the first step of the recruitment.

To evaluate the performance of the proposed MCS system, the following metrics are used.

- Average residual energy denotes the remaining battery level of users at the end of the entire sensing event.
- Average energy consumption denotes the average energy consumption of mobile user devices at the end of the sensing event.
- Platform utility: The utility of the MCS platform is the difference between the total value of sensing tasks and the total amount of rewards given to the winners through a reverse auction procedure.
- User utility is formulated as the difference between total rewards received by the winners and the total cost of sensing for the winners, which actually consists of both ordinary cost and energy cost, as reported in Section 4.4.2.
- The number of participants is used to represent the number of recruited users in a sensing campaign.
- The number of dead phones is one of the key metrics to emphasize motivating the user to participate in the MCS platform.
- The number of breakout phones denotes the number of devices that are discarded by the platform due to having their remaining battery levels below the minimum allowed level.

### 5.2. Simulation Results

The simulation results aimed at evaluating the impact of introducing sociability-orientation and energy-awareness to the baseline solution. We considered three different scenarios for the residual energy threshold ($E_{\vartheta }$) where the threshold was set to either of the following values in each scenario: 10%, 15% and 20%.

**Figure 9:**
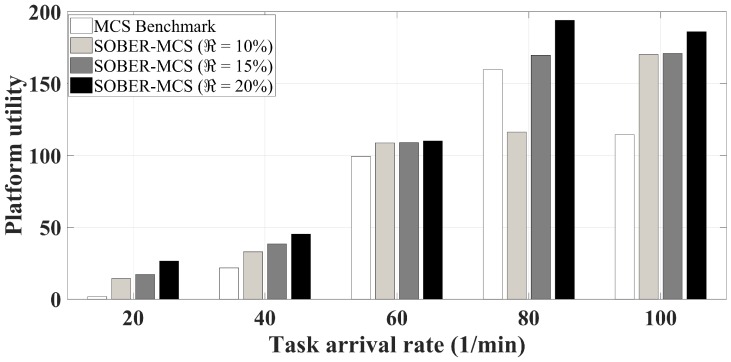
Platform utility. SOBER-MCS, Sociability-Oriented and Battery-Efficient Recruitment for Mobile Crowd-Sensing.

**Figure 10:**
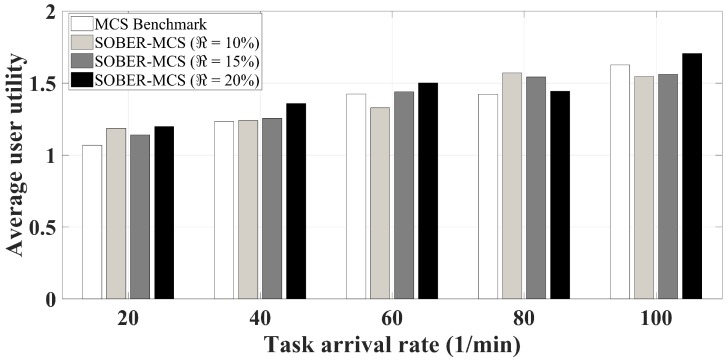
Average user utility.

Figure 9 depicts the platform utility by comparing four different scenarios: the MCS benchmark (i.e., MSensing), SOBER-MCS (Sociability-Oriented and Battery-Efficient Recruitment for Mobile Crowd-Sensing) with the residual battery level threshold of 10%, SOBER-MCS with the residual battery level threshold of 15% and SOBER-MCS with the residual battery level threshold of 20%. With the increasing task arrival rate from 20–100, the platform utility increases under each recruitment scheme as a result of the increase in the quantity (and consequently, the value) of the data. Under very lightly arriving task patterns (i.e., 20 tasks/min), SOBER-MCS with the residual energy threshold level of 10% can introduce over a 14-times improvement to the performance of MSensing. When the residual battery level threshold is set to 15%, the improvement can reach up to 20%. Finally, setting the residual energy threshold to 20% can improve the platform utility of the benchmark by >26%. These improvements follow almost a constant behavior under all sensing task arrival times. For instance, under the very heavy task arrival rate of 100 tasks/min, over 50% improvement in platform utility can be achieved when the residual battery level is set to 20%. The improvement is basically achieved by the introduction of energy-awareness (i.e., awareness of the residual battery level of the participating smartphone) to the recruitment strategy allowing one to exclude the devices that are likely to die (i.e., “breakout phones” as termed in this paper) before the completion of the sensing campaign. The only exception in the improvement of platform utility is under the case where the task arrival rate is 80 tasks/min and the residual battery level is set to 10% in the SOBER-MCS strategy. In the absence of a feasible solution to address the platform-user utility trade-off, the proposed recruitment strategy is expected to favor user utility against platform utility since the same number of tasks is covered by less participants. While this is mostly related to the uniform nature of the user distribution over the terrain, Figure 10 supports the behavior explained above with significantly higher user utility.

A closer glance at Figure 10 reveals the competent behavior of the proposed recruitment strategy, namely SOBER-MCS. As a result of the conflicting nature of the participants and the platform, it is almost inevitable to sacrifice either platform utility or user utility to introduce a significant increase in the other performance metric. However, as seen in the figure, the proposed approach does not lead to any decrease in user utility. On the contrary, under extreme load levels (i.e., 20 tasks/min and 100 tasks/min), setting the residual battery level threshold to 20% can further improve average user utility by roughly 5%. The platform-user utility trade-off is addressed through the breakout phones that participate in the sensing campaign with alarming battery levels. Exclusion of the breakout phones allows for the remaining participants to be assigned more tasks and consequently be rewarded higher than a scenario where all participants are considered in the auction regardless of their residual battery levels.

**Figure 11:**
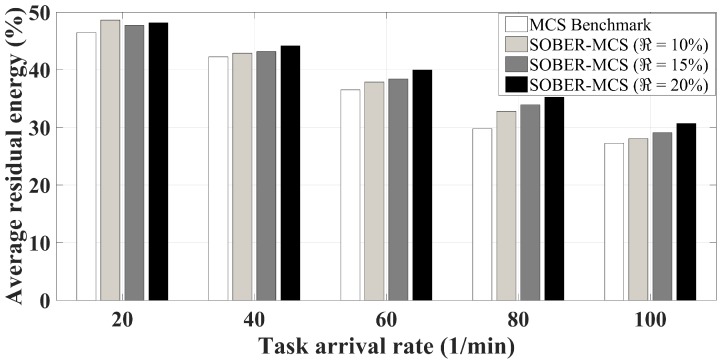
Average residual energy under SOBER-MCS with various residual battery levels.

Figure 11 illustrates the average remaining battery levels of mobile devices at the end of each event. In this study, Nexus 7 tablets with 16 WH battery capacity are used. Therefore, 100% battery level in the figures translates to 16 Wh battery power. First of all, as one can tell intuitively, heavier sensing task arrivals will drain more battery power as the recruitment schemes in the figure behave under varying sensing task arrival rates. Energy-unaware recruitment of users may end up having some of the participants’ smartphones left with fully-drained battery power. Therefore, discarding those with insufficient battery levels upfront will improve the average residual battery level at the end of the sensing campaigns. Furthermore in SOBER-MCS, users considering the energy overhead in relation to their sociability factors results in the platform being inclined toward recruiting those with higher sociability in order to avoid compensating the high energy overheads of inactive users. As expected, the highest battery drain is under heavily-arriving sensing task requests (i.e., 100 tasks/min). As can be clearly seen in the figure, SOBER-MCS improves the residual battery level as the sensing task arrival rate increases from 20 task/min–100 task/min. Under the sensing task arrival rate of 20 task/min, SOBER-MCS saves the battery of mobile devices by roughly 5% when compared to the benchmark when the residual energy threshold is set to 15% of the battery level, whereas the improvement is 3.7% when the residual energy threshold is set to 20%. Under a heavy task arrival rate (i.e., 100 tasks per min), SOBER-MCS improves the residual battery level performance of the benchmark scheme by 3%, 7% and 13% when the residual energy threshold is set to 10%, 15% and 20% of a fully-charged smartphone battery, respectively. Overall, SOBER-MCS is able to reduce the energy costs of participants in all scenarios, which is expected to result in user satisfaction and eventually incentives for more users to participate in MCS events.

**Figure 12:**
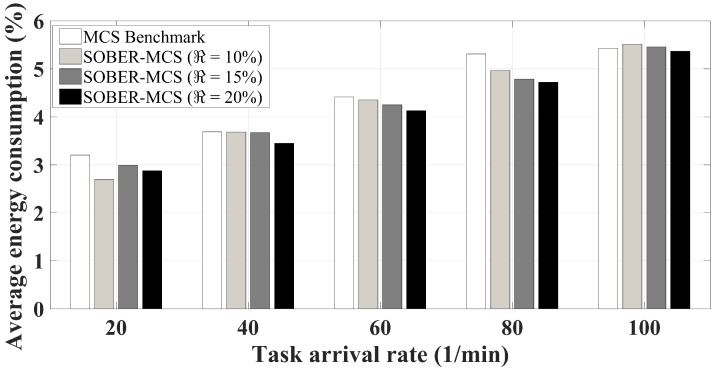
Average energy consumption.

Figure 12 is a complement to Figure 11. As the residual energy of mobile devices increases, it is intuitive to expect the average energy consumption to decrease. As seen in the figure, the average energy consumption of SOBER-MCS under all battery level thresholds for all task arrival rates is lower than that under the benchmark scheme.

**Figure 13:**
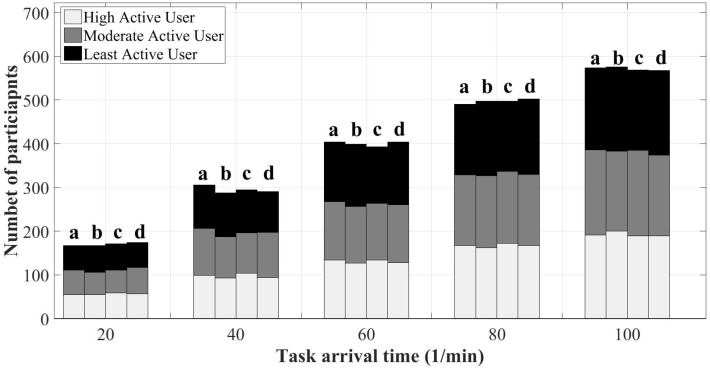
User participation in different scenarios: (**a**) mobile crowd-sensing benchmark; (**b**) SOBER-MCS with *ℜ* = 10%; (**c**) SOBER-MCS with *ℜ* = 15%; and (**d**) SOBER-MCS with *ℜ* = 20%.

Figure 13 depicts the recruitment policy of SOBER-MCS in comparison with the benchmark scheme in terms of the number of participants of different user profiles on average. As seen in the figure, the total average number of participants in all policies and under different arrival rates coincide, which means that SOBER-MCS does not impose additional cost to the platform by recruiting more users, and this behavior is independent of the value of the residual battery threshold. Instead, the algorithm attempts to make a compromise between the platform and user utilities by selecting users at the right time and by considering their availability. Thus, the user with higher probability to accept the task based on their availability undertakes the task. When a user is active (i.e., in interaction with the smartphone) at the time of sensing, the built-in sensors of the smartphone are enabled and are ready to accept the task. In this case, the user does not experience the energy overhead to start the sensing task and is more likely to be able to undertake the task. The results show that under the arrival rates of 40 task/min, 60 task/min and 100 task/min, the system recruits slightly less than the benchmark scheme. The results regarding the platform and user utility under these arrival rates comply with these as the algorithm successfully addresses the trade-off between the platform and user utilities by recruiting slightly smaller number of participants and at the same time maximizing the platform and user utilities.

**Figure 14:**
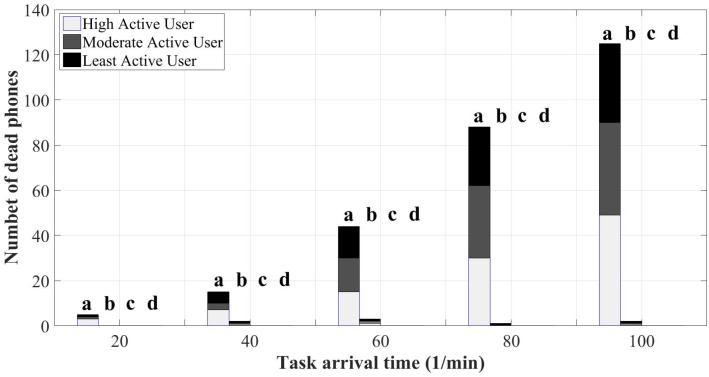
Number of dead phones: (**a**) mobile crowd-sensing benchmark; (**b**) SOBER-MCS with *ℜ* = 10%; (**c**) SOBER-MCS with *ℜ* = 15%; and (**d**) SOBER-MCS with *ℜ* = 20%.

Figure 14 illustrates the number of dead phones after the MCS event, which is an undesirable situation for the participants. in SOBER-MCS, the platform prevents from assigning sensing tasks to mobile devices that are very likely to decline accepting the task assignment due to the low battery level since by accepting the assignment, the phone may turn off before the completion of sensing. As seen in the figure, SOBER-MCS achieves a reduction in the number of dead phones such that it is a negligible value. Just for the case when the minimum residual energy is set to 10%, a small number of dead phones is experienced. However, by comparing it to the MCS benchmark, the number of dead phones even under this setting is negligible. Under all arrival rates, the results show a sharp decrease in the number of dead phones, which will further incentivize the participants to keep collaborating with the MCS platform.

**Figure 15:**
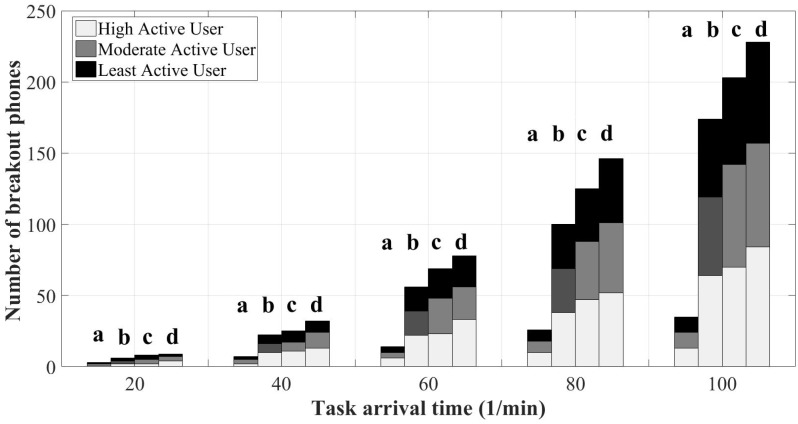
Number of breakout mobile phones: (**a**) mobile crowd-sensing benchmark; (**b**) SOBER-MCS with *ℜ* = 10%; (**c**) SOBER-MCS with *ℜ* = 15%; and (**d**) SOBER-MCS with *ℜ* = 20%.

Figure 15 presents the number of breakout mobile devices. A breakout device is a mobile phone with a battery level below the minimum residual energy. This figure actually reveals the success of SOBER-MCS in balancing the total energy consumption of the platform by excluding the users with a lower than the allowed minimum residual energy level. In this paper, the following values are considered for the minimum residual energy: 10%, 15% and 20%. As seen in the figure, the number of breakout phones grows remarkably by increasing the task arrival rate from 20 task/min to 100 task/min. When the arrival rate is 20 task/min, the proposed algorithm with the minimum residual energy of 10% doubles the number of breakout phones that are experienced under the benchmark approach. When the minimum residual energy is set to 15% and 20%, the number of breakout phones further increases, as expected. The number of breakout phones increases as the arrival rate becomes heavier since a heavier load of sensing tasks is expected to drain more energy from a smartphone battery due to the higher utilization of the built-in sensors. It is worth mentioning that the number of dead-phones is excluded from the presentation of the breakout phones. Furthermore, the number of breakout phones denotes a cumulative value such that the number of breakout phones for each auction contributes to the total value that is presented in the figure. For instance, if user *i* fails to sense the task in auction τ and in the next auction τ+1, he/she re-tries to sense the task, and he/she will be discarded one more time by the platform; thus, two breakouts will be recorded by the MCS platform for user *i*.

## 6. Conclusions

The widespread availability of smartphones, personalized devices and wearables that are equipped with highly-capable built-in sensors makes these devices viable candidates for the non-dedicated sensing paradigm of which Mobile Crowd-Sensing (MCS) is a part. MCS has recently been an emerging concept for non-dedicated sensing [3]. Built-in sensors (e.g., GPS, camera, light, accelerometer, microphone and gyroscope) in personalized smart devices are already employed to operate in various contexts such as healthcare [4,5,6], environmental/traffic monitoring and control [7,8,9,10] and public safety [43]. However, in the cloud-based Sensing as a Service (S${}^{2}$aaS) concept, the personal smart devices are considered to be significantly energy-hungry when they are integrated into a non-dedicated sensing ecosystem [16]. Therefore, cost-effective solutions in the data acquisition for MCS are emergent to lower the energy expenditures and incentivize users for participation [17].

To address this issue, in this paper, we have proposed to exploit user profiling to recruit participants in an intelligent way by assigning the task to the users with a high probability to accept the task based on their sociability (i.e., availability) and their mobile battery level. The proposed scheme is called Sociability-Oriented and Battery-Efficient Recruitment for Mobile Crowd-Sensing (SOBER-MCS). The contributions of SOBER-MCS are two-fold: (i) integration of social behavioral profiling into the recruitment process; (ii) introducing energy efficiency to the user recruitment and incentivization process with the ultimate goal of addressing platform versus user utility. These contributions have been achieved by integrating a machine intelligence component with the MCS engine to predict user availability through social activity and sociability factor features of a participant. Furthermore, by introducing a residual battery level threshold to the eligibility criterion, more devices remain in the system at the end of the sensing campaigns.

Three different classes of user profiles have been identified by the profiling engine of SOBER-MCS: highly-active user with activity more than 70% during the time window, moderately-active user with approximately 50% of activity and least active user with activity of 15% in average. The user profiles are passed to the MCS simulation framework to investigate the impact of applying defined user profiles with their settings to the MCS participants. Through simulations, we have shown that by having an additional intelligence component to recruit users based on sociability signatures and residual energy profiles, it is possible to make a compromise between platform and user utilities by selecting a user with: (i) high sociability such that the user is very likely to accept the tasks; (ii) acceptable residual battery level so that the smartphone is less likely to fail during the sensing campaign. Furthermore, SOBER-MCS increases user satisfaction by balancing the total energy consumption of smartphones by the exclusion of users with low mobile battery levels from the sensing campaign. Moreover, by recruiting highly sociable (i.e., available) users, SOBR-MCS reduces the energy consumption overhead. Consequently, the reduced overhead reduces the bids, as well as the MCS platform costs due to rewards.

We are currently investigating the impact of heterogeneous and selective sensing concepts on SOBER-MCS. Furthermore, introducing mobility prediction and GPS-less sensing to SOBER-MCS are also included in our future agenda.

## Figures and Tables

**Table 1:** Description of symbols. The notation in [41] has been partially adopted.

Symbol	Description
C	Crowd-sensing campaign
*w*	Sensing task
SS	Set of sensors | s∈SS
$SS_{s}$	sensor *s*
*u*	User *u*
U	Set of users | u∈U
W	Set of winners
*t*	Timeslot *t*
$T_{j}$	Duration of task *j*
$T_{prep}^{j}$	Time (in second) needed to prepare phone to sense the task *j*
T	Duration of the sensing task
$T_{k}$	*k*-th activity rate
*L*	Location of users and tasks
$A_{sh}^{U_{{app}_{x}}^{u}}$	Short-term social activity of user *u* on app *x*
${\mathrm{SF}}_{sh}^{U_{{app}_{x}}^{u}}$	Short-term sociability factor of user *u* on app *x*
$A_{overall}^{U_{{app}_{x}}^{u}}$	Overall social activity
${\mathrm{SF}}_{overall}^{U_{{app}_{x}}^{u}}$	Overall sociability factor
$E_{i}^{f}$	Energy factor of user *i*
$E_{w_{j}}^{u_{i}}$	Total energy consumed by user *i* to sense task *j*
$E_{t_{j}}$	Energy consumed by mobile device to run task *j*
$E_{O}^{u_{i}}$	Energy overhead consumed for user *i* to sense task
$P_{t_{j}}$	Power usage of task *j*
$P_{t_{j}}^{u_{i}}$	power usage by user *i* to sense task *j*
$P_{t_{j}}^{SS_{s}}$	Power of sensor*s* for task *j*
$P_{O_{j}}^{u_{i}}$	Overhead usage of user *i* to prepare phone to start sensing task *j*
α	Context-based parametric weight for running average calculation of activity rates
β	Context-based parametric weight for running average calculation of activity factors
*ℜ*	Residual battery level threshold
${ℜ}_{i}$	Residual battery level of user *i*

**Table 2:** Sensor power consumption [44,45].

Sensor	Power (W)
GPS	0.6173
Accelerometer	0.00165
Microphone	0.223
Light Sensor	0.025
Video camera	1.258

**Table 3:** Power overhead of different profiles.

User Profile	Power Overhead (W)
Highly-Active User	0.024
Moderately-Active User	0.04
Least Active User	0.068

**Table 4:** Simulation settings.

Parameter	Value
Terrain size	1000×1000
Sensing range	30 units
Timeslot duration	30 min
Number of users	(1000)
	Highly-active users (54%)
Initial chargeof the battery	Moderately-active users (58%)
	Least active users (63%)
Value of sensing costs	[1−10]
Task duration	Uniformly distributed in [1,5] min
Task arrival rate	(20,40,60,80,100) tasks/min
Residual energy threshold (*ℜ*)	(10%,15%,20%)
